# Dynamic Catalepsy

**Published:** 1910-05-14

**Authors:** 


### SPECIAL ARTICLE.

### DYNAMIC CATALEPSY.

Catalepsy may take one or other of two forms—the static and the dynamic. The former is usually a more lasting condition than the latter, the patients being insane enough to require treatment in institutions. The most remarkable feature of the disease is the way the sufferers strike peculiar attitudes, which are sometimes most uncomfortable, and yet which are maintained for indefinite lengths of time without apparent weariness. The dynamic form of the disease is much more violent, but also more transitory; it is of the convulsive type, and, though it is doubtless a form of insanity, really it is classed with the hysterias, and it is apt to occur in general medical practice and give rise to difficulty in diagnosis.

The following is an illustrative case:—

The patient was a lady aged thirty-eight, who was admitted to hospital in an apparently parlous condition, but whose urgent symptoms yielded so quickly that she remained in the wards but a very short time. The notes made by the house-physician are sufficiently graphic to merit quotation: "On June 5th, about 10 o'clock in the evening, I was called to a patient just brought into the hospital by a policeman, who had found her speechless in the streets. I saw a woman sitting in a chair, looking quite intelligent and coherent, although evidently in great excitement and distress. Her expression was anxious, and she looked from one to the other in an inquiring and imploring manner. Her teeth were fast clenched, and her lips parted. On being asked who she was, she shook her head. When asked what was the matter she pointed to her mouth and masseter muscles. When asked if her jaws were locked, she nodded her head. I then tried with all my force to separate them, but could not. Then I felt her cheeks over the masseters, and found these muscles contracted into hard knots, which sufficiently explained the closure of the jaws. When questioned as to the cause of the tetanus, she clenched her fist and struck her left cheek, implying that she had had a blow there. I said: 'Have you had a blow there?' She nodded her head. We asked if she could write. She shook her head, and clasped her hands in a despairing way. We then asked her if her husband had given her the blow. She nodded assent eagerly.

"By degrees, putting all sorts of questions, and getting nods or shakes of the head, as the case might be, I learned that she lived in the neighbourhood of London, that she had been struck by her husband that day at two o'clock, and that the blow was the cause of her illness; also, that she was thirty-eight years of age. All these things afterwards proved to be true. While answering our questions as well as she could, she suddenly, and without any warning, slid from her chair in a state of apparent insensibility, and would have fallen on the floor had she not been supported.

"Then commenced a series of spasms more strange and horrible than can be conceived or described. Tetanic and clonic, partial and complete, symmetrical and irregular, varied in every conceivable and inconceivable way. First she had opisthotonos, then she was thrown forwards, then she twisted round and writhed like an eel, then she would throw herself forward and raise herself into a sitting posture, then she would roll over and over, then a slow undulation or wave of spasm would pass over her from head to foot, producing different movements of her limbs as it passed down, like a dog dying of hydrocyanic acid poisoning. The tonic spasm would suddenly become clonic, and she would throw out her arms and draw them back with great force. The legs would be affected in a similar way, or drawn up to the body so that the heels were close to the buttocks, and then as suddenly thrust stiffly out. Meanwhile her face was undergoing all sorts of contortions, at one time expressing rage, at another intense fear, then a sneer, then a fixed and rigid stare. The eyes might be rolled upwards or downwards, the pupils dilated and insensible to light. Then the elevators of the upper lip and alæ of the nose would jerk, perhaps on one side or possibly on both sides at once, exposing the teeth. The depressor anguli oris would draw one corner of the mouth down on the chin, or the platysma myoides throw the skin of the neck into a state of rigidity. . . . ." This description is no exaggeration, for it is indeed quite impossible by description to give an accurate idea of the extraordinary contortions of the patient as seen by an actual onlooker.

One of the most remarkable features in the case was the rigid tonic condition of the whole muscular system. Every muscle employed in any movement became as hard as a board, and the movement was not so much executed with rapidity as with great force, and even slowness. Nevertheless, in watching the patient attentively, it was plain that the movements, varied and irregular as they were, did not partake strictly of the character of involuntary movements. They were evidently influenced by a will, but by a diseased and an ill-directed will. While these spastic movements of the muscles continued there appeared to be complete insensibility to surrounding objects, but when they ceased the patient's intellect seemed to recover itself quickly, and she could presently talk freely and collectedly. At this time she gave her history more fully; but in the midst of her narrative her eyes suddenly became fixed, she stared at the people round about her for a few moments, and then went off into another paroxysm, going through the same series of varied movements and contortions as before.

She continued in this state, alternatively in fits and quiet, for nearly two hours, and then slept.

---

On being called to a case of this kind one has to decide upon its real nature on the spot, without the help of any history on which full reliance can be placed. It is necessary to distinguish these extraordinary muscular movements from those of lockjaw, of tetanus, of strychnine poisoning, and of epilepsy. The distinction from lockjaw was here sufficiently easy. The fact of the patient coming to herself so soon, and the subsidence of the spasmodic action of the muscles, were almost sufficient to exclude that condition. The same applies to the seizures in strychnine poisoning, where, moreover, rigidity of the jaw is very uncommon. In the present case not only were the movements extremely irregular, but they bore a decided resemblance to the voluntary class, and they could not be excited at the will of the observer. On these grounds tetanus and strychnine poisoning were excluded.

At first sight, especially at the beginning of each paroxysm, the affection might seem to bear a greater resemblance to epilepsy than to any of the other forms of convulsive disease; but there were points which made it possible to decide positively that it was not epilepsy. These were the nature of the movements and the condition of the consciousness. The muscular contraction did not pass through a definite sequence from tonic to clonic, but upon the whole tended to have a continued substratum that might be termed tonic, with extraordinarily varied, combined, and irregular movements superposed upon that. The most important feature, however, was the state of the consciousness, and it was chiefly by that that it was possible to decide against the epileptic nature of the case.

In epilepsy complete coma, with total loss of consciousness, forms an essential and specific character. Convulsions form no necessary part of the epileptic paroxysm. There may be a complete absence of convulsions in epilepsy, but the insensibility is invariably present. Thus in ordinary *petit mal* there is sudden and even momentary loss of consciousness without any convulsion whatever. Such cases are not less truly examples of the epileptic paroxysm than if the most violent convulsions were also present.

In the case described, however, there was no complete loss of consciousness. There was undoubtedly an affection of consciousness, so that the patient was insensible to all ordinary external stimuli, and quite without the perception of what was going on around her, but at the same time aware of the altered state of the mental powers. In epilepsy the patient knows nothing of his mental state during the paroxysm; he is only conscious of his state before and after it—and the period of the fit is to him as if it had never existed. The nature of the movements showed that consciousness was not wholly in abeyance; they exhibited a co-ordination and a partial regularity or purposefulness which is never observed when there is complete loss of consciousness. The fingers were clutched in a steady manner as if the muscles were obeying the mandates of her will, and her body was contorted by a combined action, forming a striking contrast with the jerking movements seen in states of insensibility. The apparent insensibility in these cases depends, perhaps, upon an intense concentration of the morbid attention on one particular object. The same kind of thing in very much slighter degree is to be seen in everyday life. It is well known that when the mind is very much interested and engrossed with any particular subject or train of thought, one is very apt to take no notice of occurrences which are taking place close to, or in immediate connection with, one's person; and to be unconscious even of noises; nay, one may suffer a moderately severe pain, from a pinch for example, without being sufficiently roused from the state of reverie to direct attention to any but the one object; in fact, the mind is fixed on that one object to the complete exclusion of everything else for the time being. The state of consciousness into which such patients as the one described fall is of this nature, although very different in degree.

If, then, the disease was neither tetanus, nor strychnine poisoning, nor epilepsy, what was it? All the symptoms, especially in view of the fuller history obtained afterwards, show that it was an example of a highly developed hysterical paroxysm or fit, or, more exactly, of that peculiar and aggravated form of hysteria which is called *catalepsy*.

The history obtained from the patient and her relatives subsequently showed that she was a highly excitable, hysterical person, who had been subjected to moral and physical influences well calculated to keep up that state. She enjoyed good health until she reached the age of ten, when she experienced a sudden fright, and fell, in a fit, down the well of a staircase for the distance of two storeys. From that time until her marriage, at fifteen, she had had the fits repeatedly, generally twice a week, and sometimes oftener. Since her marriage she had had the paroxysms less frequently, but she had never gone for any long period without one.

It is perhaps noteworthy that both her paternal grandfather and a paternal uncle had fits, and the patient herself had been the mother of nine children, eight of whom died, seven of them in convulsions.

It is difficult to determine what was the precise nature of the patient's earlier fits, whether they were epileptic or hysterical. The age at which they began was more favourable to the former, for it is very unusual for a girl of ten years old to exhibit marked symptoms of hysteria. Manifestations of the hysterical condition are not common until after the appearance of the catamenia. Nevertheless the subsequent history would seem to point to the whole of the attacks having been hysterical.

The girl was persuaded to marry at the age of fifteen, the friends thinking that the attacks might thereby be mitigated or cured. She had no great affection for her husband, consequently domestic squabbles occurred very soon after their union; in some of these disagreements her husband used violence, and each time the seizures followed. At last the husband and wife agreed to separate; but circumstances obliged them to have occasional interviews, and scenes of violence were repeated almost every time. It was at one of these *rencontres* that the present attack began.

The influence of moral causes in inducing the paroxysm is very manifest in this history. Ever since she was fifteen, she stated, the slightest excitement would bring one on. It was clear that she was a woman of strong passions and violent temper. It was also noteworthy that she exhibited that peculiar appearance of countenance that has been termed the "facies hysterica," characterised by a remarkable depth and prominent fulness, with more or less thickness, of the upper lip, and also a fulness and obviously drooping condition of the upper eyelids.

Even when she was apparently well she exhibited that irritable state of spine under which hysterical patients are apt to suffer. The least pressure on a spinous process used to cause her to shrink, and to complain of a pain shooting forwards from the point pressed on; and, as is so often the case with patients of this temperament, she complained of pain from pressure in whatever part of the body it might be applied. Wherever she was touched, whether the finger came in contact with her arms, or back, or any other part of the body, she exhibited an undue degree of sensibility, and shrank.

There is yet another point about the case which may be mentioned. She was very irregular in menstruation. In the great majority of cases of hysterical affections there is found something wrong about the uterine functions; and although it cannot be laid down positively that the disease, as its name implies, depends upon this disturbance, it rarely happens that a hysterical patient has not some abnormality of the menstrual function. It is quite possible, of course, that the irregularity is the result of the hysteria rather than its cause. A large proportion of hysterical patients suffer from leucorrhœa; in others the catamenia are insufficient in quantity, or occur at irregular intervals. Sometimes the menstrual flow recurs at short intervals, and is very profuse. Perhaps there is an irritable condition of the uterus, causing almost constant pain and tenderness in the pelvic region. No one of these conditions is constantly observed, but in almost every case of hysteria there is some form of uterine disturbance.

